# Immunologic and inflammatory pathogenesis of chronic coronary syndromes: A review

**DOI:** 10.1097/MD.0000000000040354

**Published:** 2024-11-01

**Authors:** Tingting Chen, Ying Yang

**Affiliations:** a Dali University School of Clinical Medicine, Yunnan, China; b Department of Cardiology, The First Affiliated Hospital of Dali University, Yunnan, China.

**Keywords:** chronic coronary syndrome, immune, inflammatory, pathogenesis

## Abstract

Chronic coronary syndrome (CCS) is a major cause of progression to acute coronary syndrome. Due to its insidious onset and complex etiology, this condition is often underestimated and insufficiently recognized, and traditional interventions for risk factors do not effectively control the disease progression. Current research suggests that immune and inflammatory pathways contribute to atherosclerosis and its clinical complications, thereby triggering the progression of CCS to acute coronary syndrome. This article primarily reviews the possible mechanisms of immune and inflammatory responses in CCS, with the aim of providing references for the diagnosis, treatment, and prevention of CCS.

## 1. Introduction

Chronic coronary syndrome (CCS) refers to clinical syndromes of coronary artery disease (CAD) other than acute coronary syndromes (ACS).^[1]^ Although the latest guidelines in 2023 updated the disease terminology to chronic coronary disease (CCD),^[2]^ the definitions of “CCD” and “CCS” and the patient populations they cover are essentially the same, the following 5 types of CCD are common in the clinic: (1) patients discharged after admission for an ACS event or after coronary revascularization procedure and after stabilization of all acute cardiovascular issues; (2) patients with left ventricular systolic dysfunction and known or suspected CAD or those with established cardiomyopathy deemed to be of ischemic origin; (3) patients with stable angina symptoms medically managed with or without positive results of an imaging test; (4) patients with angina symptoms and evidence of coronary vasospasm or microvascular angina; (5) patients diagnosed with CCD based solely on the results of a screening study, and the treating clinician concludes that the patient has coronary disease. The stability of CCS in the non-acute phase is relative, and in most cases, the disease is progressing and worsening, significantly increasing the risk of acute coronary events.^[3]^ The new definition in the guidelines reflects an evolving understanding of the CAD spectrum in clinical practice, which helps to better inform and guide clinical work.

The pathophysiological core of CCS involves the accumulation of atherosclerotic plaques and alterations in coronary circulation function.^[1,3,4]^ Almost half of a population can harbor subclinical atherosclerosis without a high burden of traditional risk factors.^[5]^ Thus, active lifestyle interventions and control of conventional risk factors, some patients still progress to ACS. Ongoing research is concentrating on the impact of immune and inflammation-related factors in the onset of CCS, venturing beyond the conventional risk factors.^[6–8]^ This review aims to elucidate the immunological and inflammatory mechanisms involved in CCS progression, with the aspiration of slowing or even reversing disease progression.

## 2. Evidence for immune mechanisms in CCS

There is growing evidence that innate and adaptive immunity play a crucial role in the pathophysiology of AS and that atherosclerotic lesions are a hallmark of CCS.^[9,10]^ Although they have different mechanisms of action, synergy between them is essential for a fully effective immune response.^[11]^ Local and systemic immune responses are key driving factors in atherosclerosis, contributing to thrombosis formation and myocardial remodeling following acute myocardial ischemia.^[7,12]^ Studies targeting immunity may provide potential new prognostic markers and treatments for patients with CCS.

### 2.1. Mechanisms of innate immunity

The involvement of multiple cells of the innate immunity, such as macrophages, dendritic cells (DCs), monocytes, mast cells, and neutrophils, is linked to the severity of atherosclerosis.^[13]^ Plaque monocytes differentiate into macrophages and monocyte-derived DCs that present antigens to T cells.^[14]^ Monocyte-derived macrophages initiate lipoprotein uptake and clearance, leading to the formation of lipid-rich foam cells.^[15]^ Increased oxidative stress and accumulation of modified lipoproteins in the arterial wall may overwhelm or reconnect macrophage metabolism.^[16]^ This activates a cascade of inflammatory cytokines and chemokines, resulting in the infiltration and activation of pro-atherosclerotic immunocytes.

Both conventional DCs and plasmacytoid DCs have been found in the arteries of both mice and humans, as well as in lesions associated with atherosclerosis.^[17]^ These cell types, conventional DCs and plasmacytoid DCs, can influence the progression of atherosclerosis in mice by modulating T-cell activation and adaptive immune responses in an antigen-dependent manner. However, research regarding the role of DCs in human atherosclerosis is still at an exploratory stage, with their functional contributions still awaiting further exploration.^[18]^

### 2.2. Mechanisms of adaptive immunity

Adaptive immunity is a key regulator of atherosclerosis.^[19]^ Adaptive responses are mainly based on antigen-specific receptors expressed on the surface of T and B lymphocytes.^[20]^ Subsets of CD4+ T cells can variably affect the progression of atherosclerosis through mechanisms of immune activation or suppression, or by assisting B cells in antibody production.^[21]^ Numerous pieces of evidence, such as genetic anomalies in T-bet, IFNγ or its corresponding receptor, as well as the treatment with exogenous IFNγ, have confirmed that TH1 cells play a pro-atherosclerotic role in advancing lesion formation and contributing to plaque instability.^[22]^ B cells can secrete a variety of cytokines that have a pronounced effect on inflammation. For example, pro-atherosclerotic IRA-B cells secrete GM-CSF to drive myeloid activation and induce pro-atherosclerotic TH1 immunity.^[23]^

An enhanced comprehension of how innate immunity cells together with adaptive immunity cells function in atherosclerosis has led to numerous research endeavors and clinical studies aimed at evaluating the viability of immunomodulatory treatments for cardiovascular diseases. Tolerogenic vaccination has surfaced as a potential approach to enhance antigen-specific, anti-atherogenic immune responses, both humoral and cell-mediated, in the context of atherosclerosis.^[24]^

### 2.3. Immunomodulating therapies

Both innate and adaptive immune parameters of the immune response are involved in the initiation, progression, and eventual thrombotic complications of atherosclerosis.^[22]^ After activation of the innate immune response, an adaptive response is initiated that further promotes atherosclerotic plaque formation.^[22]^This understanding provides a new target for reducing the pro-inflammatory environment in atherosclerosis.

Fredman et al used nanoparticles containing the pro-resolving peptide Ac2-26 to activate the membrane-bound protein A1 receptor in myeloid cells and stabilize advanced atherosclerotic lesions.^[25]^ Another study administered the potent polarizer IL-13 to atherosclerotic mice to induce macrophage M2 status, showing reduced plaque inflammation and atherosclerotic burden.^[26]^ In addition, Geng et al developed an immunomodulatory approach designed to reprogram monocytes for the treatment of atherosclerosis. They found that knockout of TRAM in ApoE^−/−^ mouse monocytes reduced the development of atherosclerotic plaques.^[27]^

These studies allow us to modulate the immune aspects of the pathogenesis of arterial diseases to treat and ultimately prevent them. However, it is currently unclear whether targeted suppression of immune responses will reduce the incidence of cardiovascular events. We need to focus on the current targeted pathways, as well as those pathways that can be easily targeted by existing medications, in order to develop better therapies and alleviate the burden of the disease.

## 3. Evidence of inflammation in CCS

The central role of inflammation in the progression of coronary disease is well recognized.^[28,29]^ Systemic inflammation might further accelerate the progression of atherosclerotic disease and increase the risk of further cardiovascular events.^[30,31]^ Several inflammatory pathways lead to the progression of atherosclerotic burden and plaque instability, commonly referred to as residual inflammatory risk.^[32]^ Measuring circulating levels of pro-inflammatory mediators, such as inflammatory cytokines and acute phase proteins, is a widely used strategy to quantify residual inflammatory risk.

### 3.1. Role of inflammatory mediators

#### 3.1.1. Role of inflammatory cytokines

IL-18 works synergistically with IL-12 to trigger the secretion of IFN-γ through macrophages,^[33]^ T cells,^[34,35]^ and NK cells,^[36]^ stimulating inflammatory responses. The IL-12–IL-18–T-bet–IFN-γ cascade is a strong pro-inflammatory stimulus that can promote and increase the development of lesion development, and AS.^[37]^

Macrophages expressing pro-inflammatory cytokines can enhance the overexpression of MMPs involved in plaque instability.^[38]^ MMPs directly or indirectly affect actions of various cytokines such as IFN-γ, TGF-β, IL-1, and TNF-α, and participate in inflammation and repair processes. The pro-inflammatory factors IL-1 and TNF-α activate a series of MMPs in vascular cells, including MMP-1, -3, -8, and -9.^[39,40]^

RANTES is a cytokine that selectively chemically attracts T cells, eosinophils, NK cells, and monocytes. The RANTES leads to endothelial dysfunction of vascular inflammatory cell recruitment, atherosclerotic plaque and neointima formation.^[41,42]^ RANTES is preserved in α-granules of the platelets and aggregates on the damaged EC surface after platelet activation, promoting the atherogenic recruitment of monocytes, which may aggregate the development of atherosclerotic plaque.^[43]^

IL-6 is a unique pleiotropic cytokine, which may play a role in promoting and anti-atherosclerosis in the formation and progression of atherosclerosis. The pro-atherosclerotic effect is to induce the proliferation of vascular SMC,^[44]^ as well as EC^[45]^ and platelet activation,^[46]^ while the protective effect of atherosclerosis includes the reduction of plasma low-density lipoprotein through the overexpression of low-density lipoprotein receptor.^[47]^

IL-1 can enhance gene amplification in cells that play a role in atherosclerosis. Cells in atherosclerosis produce IL-1 when exposed to inflammatory stimuli.^[48]^ IL-1 interferes with myocardial contractile function. IL-1 can promote ischemia-reperfusion injury and extensive cardiac remodeling after experimental myocardial infarction.^[49]^

IL-27 signaling is mediated through the signal transduction and transcriptional activation factors STAT 1 and STAT 3 pathways.^[50]^ STAT 1 and STAT 3 molecules are phosphorylated by the Janus kinase-signal transducer and activator of transcription (JAK-STAT) system and activated by the p38 mitogen-activated protein kinase (P38-MAPK) system, inducing a series of effects,^[51–53]^ promoting the differentiation of initial CD4+ T cells into Th1 cells, and facilitating inflammation.^[54,55]^

ET-1 induces endothelial dysfunction in the coronary circulation and promotes the formation of atherosclerotic plaques through several mechanisms, including decreased NO pathway activity, increased oxidative stress and inflammation, and interference with glucose and lipid metabolism. ET-1 enhances the formation of reactive oxygen species clusters and local oxidative stress, which can have deleterious effects on the vessel wall through a Ras-dependent mechanism.^[56]^ Oxidative stress accompanied by local inflammation is an important mediator of atherosclerotic plaque formation, progression and rupture.^[57]^

#### 3.1.2. Role of inflammatory acute phase proteins

High-sensitivity C-reactive (hs-CRP) is an acute phase protein that can enter the bloodstream with the release of inflammatory cytokines, causing vascular damage. At the same time, it can promote the aggregation of monocytes at the site of injury, thereby accelerating the progression of atherosclerosis.^[58,59]^ Previous studies have found that hs-CRP is an important predictor of adverse cardiovascular events in patients with CCS.^[60,61]^ Whether treated with medication alone or undergoing PCI treatment, elevated hs-CRP levels in patients with CCS or unstable angina also increase the incidence of adverse cardiovascular events.^[62,63]^

Fibrinogen is also an acute phase protein that reflects the severity of inflammation. Its degradation products can stimulate the growth and migration of leukocytes, as well as activate vascular endothelial cells, promoting the high expression of adhesion molecules. This results in endothelial dysfunction, leading to plaque rupture and embolism.^[64,65]^ In the PROCAM study, fibrinogen is also an independent risk factor for CAD.^[66]^

### 3.2. Targeted therapy for inflammation

Inflammation is a key factor in the development and progression of atherosclerosis and is a significant constituent of residual risk pertaining to cardiovascular disease.^[67]^ Other anti-inflammatory interventions for atherosclerosis include inhibition of inflammatory bodies.^[68]^ The study confirmed that the main pathway for the increased risk of residual inflammation is activation of the nucleotide-binding oligomerization structural domain-like receptor protein 3 inflammasome, which ultimately activates its downstream signaling molecules IL-1β, IL-6, and hs-CRP.^[69]^ The growing interest in the CAD inflammation hypothesis has led to the evaluation of new drugs, such as anti-IL-1β targeted drugs, which already proved to be safe and effective in other cardiovascular diseases.^[70,71]^ Because of these anti-inflammatory effects, recent studies have suggested that colchicine can be used for anti-inflammatory treatment in coronary heart disease.

Inflammation plays a crucial role in both the development and progression of atherosclerosis, serving as a significant contributor to the ongoing risk associated with cardiovascular disease.

When canakinumab works by selectively inhibiting IL-1 β, colchicine works through a mechanism believed to be associated with inhibition of inflammasome and neutrophil recruitment and adhesion,^[72,73]^ reduces the risk of recurrent myocardial infarction, stroke or death from cardiovascular causes by 15% in patients with CCS or after myocardial infarction.^[74]^

Studies have shown that in patients with CAD, most have received proven secondary preventive treatment, and the incidence of cardiovascular events with low-dose colchicine is significantly lower than that with placebo.^[72,75,76]^ In other words, colchicine improved patient outcome in CCS regardless of the intensity of systemic inflammation.^[77]^ Although canakinumab has been shown to reduce the incidence of cardiovascular events after myocardial infarction, its high price and potential for increased infection limit its widespread use.^[78]^

The CANTOS trial provided evidence that inflammation plays a causal role in the pathogenesis of cardiovascular disease and related complications and that interventions to reduce inflammation may reduce the risk of cardiovascular events.^[74]^ In 2023, the U.S. Food and Drug Administration approved Lodoco (colchicine, 0.5 mg tablets) for the treatment of atherosclerotic cardiovascular disease, making it the first anti-inflammatory drug approved for cardiovascular disease.

Therefore, targeting inflammation is an effective strategy for reducing the risk of cardiovascular events, even for patients who have already received optimal drug therapy for CAD and have achieved traditional risk factor targets.^[79]^ Based on this, targeting inflammation has gradually become a new area in the treatment of coronary heart disease.

## 4. Interaction of immune and inflammatory mechanisms

In recent years, inflammation-based markers composed of inflammatory and immune cells have been found to not only reflect the stability of atherosclerotic plaques but also serve as important predictive indicators for the risk factors of stable coronary heart disease. They can effectively assess the body’s inflammatory status and the balance of the immune system. In previous studies, various IBMs such as systemic immune-inflammation index,^[80]^ CRP-to-albumin ratio,^[81]^ platelet-to-lymphocyte ratio,^[82]^ neutrophil-to-lymphocyte ratio,^[83]^ monocyte-to-lymphocyte ratio,^[84]^ monocyte-to-high-density lipoprotein cholesterol ratio (MHR),^[82]^ triglyceride-glucose index,^[85]^ uric acid/albumin ratio,^[83]^ and pan-immune-inflammation^[86]^have been identified as independent predictors of disease progression in coronary heart disease.

We now recognize that inflammation and immune responses can either positively promote disease or negatively modulate deleterious effects and promote regression or repair of lesions.^[87]^ The immune system influences the state of inflammatory cells by transforming them into pro-inflammatory or anti-inflammatory functional units, and by guiding interactions between different immune and inflammatory cells.^[88]^ The pathways between different cells can regulate various aspects of plaques, leading to their disruption and providing a focus for thrombosis formation.^[89]^ At the same time, immune activation can sustain local inflammatory responses, and the production of matrix metalloproteinases can degrade the fibrous cap covering atherosclerotic plaques, resulting in necrotic core formation, which adds another dimension to their ability to regulate atherosclerotic complications.^[90]^

Therefore, we have reason to believe that atherosclerosis functions as a long-lasting inflammatory disease that includes autoimmune factors,^[91]^ and inflammation and immune mechanisms can link traditional atherosclerotic risk factors and provide new diagnostic and therapeutic insights for clinical interventions in patients with CCS.

## 5. Conclusion and future perspectives

In summary, inflammation and immunity do not replace or challenge traditional risk factors, but rather provide further mechanistic explanations that link these risk factors to alterations in the behavior of the vascular wall cells that give rise to disease and its complications. Thus, the notion that immunity and inflammation can promote atherosclerosis formation or trigger atherosclerotic events does not challenge the notion that cholesterol plays a key role in the development of the disease; rather, further exploration of these avenues may address the unacceptable residual risk burden that persists beyond traditional risk factor management. Inflammatory and immune mediators also affect coagulation and fibrinolysis, providing another dimension to their ability to regulate atherosclerotic complications. It is anticipated that in the near future, with more evidence emerging, the suppression of inflammation or immunity is likely to become the fourth cornerstone of CAD treatment, alongside lowering low-density lipoprotein cholesterol, inhibiting platelet aggregation, and controlling other risk factors.

CCS is a progressive form of CAD, and we must fully recognize the complexity of inflammation and immune mechanisms. However, various inflammation-based markers that are derived from inflammation and immune mechanisms, which are clinically relatively simple and easily accessible, are indicative of the development and severity of such diseases. Therefore, we should actively seek specific inflammation markers for the early detection, early diagnosis, and early treatment of these diseases. Whether through antibody therapies, vaccination, or targeted inhibition of adaptive pro-inflammatory responses, treatment strategies aimed at reducing atherosclerosis formation should be approached with caution. Finally, we hope to continue obtaining intriguing data related to the immune and inflammatory mechanisms associated with CCS to prevent the exacerbation of the already prevalent mortality and morbidity rates.

## Author contributions

**Conceptualization:** Tingting Chen.

**Data curation:** Tingting Chen.

**Investigation:** Tingting Chen.

**Methodology:** Ying Yang.

**Resources:** Tingting Chen.

**Supervision:** Ying Yang.

**Writing – original draft:** Tingting Chen.

**Writing – review & editing:** Tingting Chen, Ying Yang.
